# 3-Hydroxyphthalic Anhydride-Modified Chicken Ovalbumin as a Potential Candidate Inhibits SARS-CoV-2 Infection by Disrupting the Interaction of Spike Protein With Host ACE2 Receptor

**DOI:** 10.3389/fphar.2020.603830

**Published:** 2021-01-14

**Authors:** Taizhen Liang, Jiayin Qiu, Xiaoge Niu, Qinhai Ma, Chenliang Zhou, Pei Chen, Qiao Zhang, Meiyun Chen, Zifeng Yang, Shuwen Liu, Lin Li

**Affiliations:** ^1^ Guangdong Provincial Key Laboratory of New Drug Screening, Guangzhou Key Laboratory of Drug Research for Emerging Virus Prevention and Treatment, School of Pharmaceutical Sciences, Southern Medical University, Guangzhou, China; ^2^ School of Pharmaceutical Science, Zhejiang Chinese Medical University, Hangzhou, China; ^3^ Department of Special Medical Service Center, Zhujiang Hospital, Southern Medical University, Guangdong, China; ^4^ State Key Laboratory of Respiratory Disease, National Clinical Research Center for Respiratory Disease, Guangzhou Institute of Respiratory Health, The First Affiliated Hospital of Guangzhou Medical University, Guangdong, China

**Keywords:** SARS-CoV-2, 3-hydroxyphthalic anhydride-modified chicken ovalbumin, spike, fusion inhibitor, angiotensin-converting enzyme 2

## Abstract

The global spread of the novel coronavirus SARS-CoV-2 urgently requires discovery of effective therapeutics for the treatment of COVID-19. The spike (S) protein of SARS-CoV-2 plays a key role in receptor recognition, virus-cell membrane fusion and virus entry. Our previous studies have reported that 3-hydroxyphthalic anhydride-modified chicken ovalbumin (HP-OVA) serves as a viral entry inhibitor to prevent several kinds of virus infection. Here, our results reveal that HP-OVA can effectively inhibit SARS-CoV-2 replication and S protein-mediated cell-cell fusion in a dose-dependent manner without obvious cytopathic effects. Further analysis suggests that HP-OVA can bind to both the S protein of SARS-CoV-2 and host angiotensin-converting enzyme 2 (ACE2), the functional receptor of SARS-CoV-2, and disrupt the S protein-ACE2 interaction, thereby exhibiting inhibitory activity against SARS-CoV-2 infection. In summary, our findings suggest that HP-OVA can serve as a potential therapeutic agent for the treatment of deadly COVID-19.

## Introduction

Novel coronavirus disease 2019 (COVID-19), a respiratory disease caused by severe acute respiratory syndrome coronavirus 2 (SARS-CoV-2), continues to spread worldwide (Kuan et al., 2016; Sharma et al., 2020; Zhou et al., 2020). The World Health Organization (WHO) has characterized the epidemic situation of SARS-CoV-2 as a “Public Health Emergency of International Concern” (Song and Karako, 2020; Wang et al., 2020), which has aroused widespread concern in the world and has brought significant threats to international health and social stability, thus calling for the development of highly effective therapeutics and prophylactics (Kampf et al., 2020; Wu et al., 2020). SARS-CoV-2 is an enveloped positive-sense, single-stranded RNA virus and belongs to the β-coronavirus genus, which shares high genetic sequence identity with severe acute respiratory syndrome coronavirus (SARS-CoV) and bat SARS-like coronavirus (SL-CoV) (Tian et al., 2020). Notably, SARS-CoV-2 has lower pathogenicity and higher transmissibility than SARS-CoV, which may explain the severity of the epidemic (Lai and Cavanagh, 1997; Peiris et al., 2004; Li et al., 2020).

Similar to other two coronavirus strains, including SARS-CoV and Middle East respiratory syndrome coronavirus (MERS-CoV), cell entry of SARS-CoV-2 is the first step of cross-species transmission. SARS-CoV-2 contains four important structural proteins: the spike (S), envelope (E), membrane (M), and nucleocapsid (N) proteins. The S, E, and M proteins promote virus assembly and entry into host cells, and the N protein is needed for RNA synthesis (Li, 2016; Schoeman and Fielding, 2019; Ortiz-Prado et al., 2020). The S protein on the surface of SARS-CoV-2 cells is composed of a receptor-binding unit S1 and a membrane-fusion unit S2 (Rota et al., 2003; Walls et al., 2020). First, S1 can bind to the cellular surface receptor angiotensin-converting enzyme 2 (ACE2) through its receptor-binding domain (RBD) to initiate infection (Hoffmann et al., 2020). Second, S2 helps viral genomes enter host cells by fusing the host cell and viral membranes. The interactions between the S protein and the ACE2 receptor play an important role in viral entry into host cells (Wu et al., 2012; Huo et al., 2020a; Tai et al., 2020). Therefore, it might be a potential approach to screen special antibodies or small-molecule inhibitors for blocking the RBD and ACE2 interaction and preventing virus infection (Chu et al., 2008; Du et al., 2009; Huo et al., 2020b).

Many molecules targeting the S protein have been found to be effective *in vitro*. The fusion inhibitors EK1C4 (Xia et al., 2020), IPB02 (Zhu et al., 2020) and nelfinavir mesylate (Viracept) (Musarrat et al., 2020) potently inhibit SARS-CoV-2 S protein-mediated cell-cell fusion and pseudovirus infection. SARS-CoV and SARS-CoV-2 cellular entry can be blocked by the protease inhibitor camostat mesylate and the cathepsin L inhibitor E-64d (Hoffmann et al., 2020). Apilimod, a potent inhibitor of phosphatidylinositol 3-phosphate 5-kinase (PIKfyve), can significantly reduce the entry of SARS-CoV-2 S pseudovirus into 293/hACE2 cells *via* early endosomes in a dose-dependent manner (Hoffmann et al., 2020; Kang et al., 2020). Several SARS-CoV-specific neutralizing antibodies such as CR3022, m396 and S309 have been further demonstrated to interact with SARS-CoV-2 S protein. However, only S309, rather than CR3022 and m396, showed potent cross-neutralizing activity on SARS-CoV-2, indicating that subtle difference in the RBD of SARS-CoV-2 and SARS-CoV may limit the cross-reactivity of SARS-CoV-specific neutralizing antibodies with SARS-CoV-2 (Hussain et al., 2020; Tian et al., 2020). Until now, there are still some disadvantages to these antiviral agents. They generally produce toxic responses, have a short half-life and cause acute side effects. Therefore, these weaknesses might affect their clinical use, and there is an urgent need to find new and effective therapeutics for the treatment of COVID-19.

Our previous studies have reported that several kinds of viruses, including human immunodeficiency virus (HIV), human papillomavirus (HPV), respiratory syncytial virus (RSV), and novel human coronavirus MERS-CoV, can be inhibited at the viral entry step by anhydride-modified proteins (Li et al., 2010a; Zhao et al., 2013; Hua et al., 2019). Furthermore, one kind of anhydride-modified bovine protein, β-lactoglobulin (β-LG), was clinically applied to treat HPV infection (Hua et al., 2019). Therefore, we decided to investigate whether anhydride-modified proteins could be utilized as anti-SARS-CoV-2 antivirals. In particular, 3-hydroxyphthalic anhydride-modified OVA is convenient for anhydride modification, which is isolated from chicken eggs and less expensive than rabbit serum albumin (RSA), which is purified from animal sera. Luckily, due to the broad-spectrum antiviral effect of anhydride-modified proteins, we screened the anti-SARS-CoV-2 activity of different anhydride-modified proteins and found a potential candidate, HP-OVA, which is highly effective in inhibiting infection by blocking the RBD and ACE2 interaction. In this study, we verify the entry-inhibitory activity of HP-OVA against SARS-CoV-2, and the results suggested that HP-OVA could be developed as a novel viral entry inhibitor used to prevent and treat SARS-CoV-2 infection.

## Materials and Methods

### Cell Lines and Plasmids

The human embryonic kidney cell line 293T (HEK-293T), African green monkey kidney cell line Vero E6 and human hepatoma Huh 7 cell lines were obtained from the American Type Culture Collection (ATCC). HEK-293T cells stably expressing human ACE2 (293T/ACE2) were established by our laboratory. All of these cells were cultured in Dulbecco’s modified Eagle’s medium (DMEM, Gibco) supplemented with 10% fetal bovine serum (FBS, Capricorn Scientific, Germany), 100 units/ml penicillin, 100 μg/ml streptomycin and 2% l-glutamine (Gibco).

The envelope-expressing plasmids SARS-CoV-2-S (*pcDNA3.1-SARS-CoV-2-S* and *pAAV-IRES-EGFP-SARS-CoV-2-S*) and *pcDNA3.1-SARS-CoV-S* were kindly provided by Dr Shibo Jiang (Fudan University, China). The plasmid *pAAV-IRES-EGFP* was purchased from Hedgehogbio Science and Technology Ltd (Shanghai, China). The luciferase reporter-expressing HIV-1 backbone *pNL4-3.Luc.R* ^*−*^ *E* ^*−*^ plasmid was maintained in our laboratory.

### Chemical Modification of OVA

The modified protein HP-OVA was prepared using a previously described (Li et al., 2011). Briefly, OVA (final concentration, 20 mg/ml in 0.1 M phosphate) was treated with hydroxyphthalic anhydride (HP) (1.19 M in dimethylformamide) by the addition of five aliquots at 12 min intervals, while the pH was adjusted to 8.5 with 1 M NaOH after each mixing. The mixture was kept for 1 h at room temperature and then extensively dialyzed against phosphate-buffered saline (PBS) and filtered through 0.45 μM msyringe filters (Gelman Sciences, Ann Arbor, MI). Protein concentrations were measured by a bicinchoninic acid (BCA) protein assay reagent kit (Thermo Fisher Scientific, USA). To quantitate the lysine residues in OVA and HP-OVA, 2,4,6-Trinitrobenzene Sulfonic Acid (TNBS) treatment was applied as previously described (He et al., 2011).

### Cytopathic Effect (CPE) Inhibition Assay on Live SARS-CoV-2 Infection

To assess the inhibitory activity of HP-OVA against infection by live SARS-CoV-2, 100 50% Tissue Culture Infectious Dose (TCID_50_) of SARS-CoV-2 was incubated with Vero E6 cells (2 × 10^5^/ml) at 37°C for 2 h. After 2 h post-infection, the culture supernatants were discarded and HP-OVA at graded concentrations was added to Vero E6 cells for three days. Then, the CPE was detected by fluorescence microscopy, and the 50% inhibitory concentration (IC_50_) was calculated by the Reed-Muench method or GraphPad Prism 5.0 software. Remdesivir was used as a positive control.

### Luciferase Assay on Pseudotyped SARS-CoV-2 Infection

The infectivity of pseudotyped SARS-CoV-2 and SARS-CoV on target cells was determined by a single-cycle infection assay as described previously (Yin et al., 2018). To produce pseudovirions, 293T cells were co-transfection with a plasmid expressing the S protein of SARS-CoV-2 or SARS-CoV (pcDNA3.1-SARS-CoV-2-S or pcDNA3.1-SARS-CoV-S) and a backbone plasmid (pNL4-3.Luc.R-E-) that encodes an Env-defective, luciferase reporter-expressing HIV-1 genome. The cell supernatants containing the released virions were harvested at 48 h post-transfection, passed through a 0.45 μm filter and frozen at −80°C.

To detect the inhibitory activity of HP-OVA on pseudotyped SARS-CoV-2 and SARS-CoV infection, target cells (293T/ACE2 and Vero E6) were seeded into 96-well plates at a density of 10^4^ cells per well. After overnight incubation, a series of dilutions of the compound were mixed with an equal volume of pseudovirus, and the mixture was transferred to the cells. Twelve hours after infection, the culture medium was refreshed, and then, the cells were incubated for an additional 48 h, followed by washing the cells with PBS, lysing the cells with 50 μl of lysis reagent (Promega) per well on a microperforated plate oscillator for 15 min, and transferring 30 μl of the cell lysates to 96-well Costar flat-bottom luminometer plates (Corning Costar) for the detection of relative light units using a Firefly Luciferase Assay Kit (Promega, Madison, WI). The IC_50_ was calculated as the final concentration of HP-OVA that caused a 50% reduction in relative luminescence units (RLUs) compared to the level of the virus control subtracted from that of the cell control.

### SARS-CoV-2 S-Mediated Cell-Cell Fusion Assay

HEK-293T cells were transfected with pAAV-IRES-EGFP or pAAV-IRES-SARS-CoV-2-S-EGFP as the effector cells by PolyJet^TM^ DNA *in vitro* Transfection Reagent (SignaGen, USA). Huh 7 cells/Vero E6 cells (1 × 10^4^) expressing ACE2 receptor were incubated in 96-well plates at 37°C for 5 h followed by the addition of 293T/EGFP or 293T/SARS-CoV-2-S/EGFP cells with or without compounds. After co-culture at 37°C for 12 h, three fields in each well were randomly selected to count fused and unfused cells under an inverted fluorescence microscope (Nikon Eclipse Ti-S). The percent inhibition of cell-cell fusion was calculated using the following formula, as described elsewhere [1 − (E − N)/(P − N)] × 100%. “E” represents the percentage of cell-cell fusion in the experimental group. “P” represents the percentage of cell-cell fusion in the positive control group, where 293T/SARS-CoV-2- S/EGFP cells were used as effector cells to which no compound was added. “N” represents the percentage of cell-cell fusion in the negative control group, in which 293T/EGFP cells were used as effector cells. The IC_50_ was calculated using CalcuSyn software. Samples were tested in triplicate, and all experiments were repeated twice.

### Western Blot Analysis

HE-K293T cells were transfected with 2 μg of plasmids encoding the SARS-CoV-2 S protein or ACE2 using polyethylenimine (PEI, Sigma). After 48 h, the cells were collected and lysed in RIPA buffer (50 mM Tris-HCl (pH 7.5), 150 mM sodium chloride, 1 mM EDTA, 1% Triton X-100, 0.25% sodium deoxycholate, 0.1% SDS) containing 1 × protease and phosphatase inhibitor cocktail (Merck Calbiochem, Darmstadt, Germany). Then, the cells were incubated on ice for 10 min, followed by centrifugation at 12,000 × g for 10 min at 4°C. The supernatant was collected as a whole protein extract. Total protein was quantified by a BCA Protein Assay Kit (Thermo Fisher Scientific, Carlsbad, CA). The protein extract was quantified prior to being denatured by the addition of a loading buffer (0.313 M Tris-HCl (pH 6.8), 10% SDS, 0.05% bromophenol blue and 50% glycerol), followed by denaturation at 100°C for 10 min. Then, 50 μg of total protein was electrophoresed for 1.5 h on a 10% polyacrylamide gel to separate the proteins, transferred onto PVDF membranes (Roche, Indianapolis, IN, USA), and co-incubated with an anti-SARS-CoV-2 spike antibody (40150-R007, Sino Biological, China) or ACE2 antibody (#4355, CST) at 4°C overnight and secondary antibodies conjugated to horseradish peroxidase (HRP). Protein bands were detected by chemiluminescence using an ECL kit (Millipore).

### Flow Cytometric Analysis

HEK-293T cells were transfected with 2 µg of plasmids encoding the SARS-CoV-2 S protein using PEI. Forty-eight hours later, the cells were detached by using PBS with 1 mM EDTA. After washing, the cells were incubated with PBS containing 10% goat serum (PBS-GS) at 4°C for 1 h before being treated with HP-OVA or OVA. After incubation at 4°C for 1 h, cells were washed three times with PBS-GS, and then polyclonal rabbit anti-OVA antibody (1:1,000 dilution) (Sigma) was added to the cells for 1 h on ice, followed by being incubated to Alexa Fluor 488-conjugated goat anti-rabbit IgG (1:10,000) (Abcam) for 1 h. The cells were washed and resuspended in 400 μl of PBS-GS buffer, and then analyzed by flow cytometry. Unmodified OVA was used as a negative control.

### Enzyme-Linked Immunosorbent Assay (ELISA)

ELISA was performed to identify the interaction of HP-OVA and the SARS-CoV-2 S protein (RBD) or ACE2 protein. Briefly, wells of 96-well polystyrene microplates were coated with 1 μg/mL S protein (RBD) (Sino Biological, China) or ACE2 protein (Invitrogen, Carlsbad, CA) in 0.01 M Tris buffer (pH 8.8) at 4°C overnight. Here, a bovine serum albumin (BSA) was used as an irrelevant coating protein antigen control. After washing with PBS-T three times, the wells were blocked for 2 h at 37°C with 5% BSA. Various concentrations of HP-OVA were added to the wells for 2 h at 37°C. After washing with PBS-T, the samples were incubated with a goat anti-OVA antibody (Sigma) for 1 h and then incubated with an HRP-labeled goat anti-mouse antibody for 1 h at 37°C. After color development, the optical density (OD) value at 450 nm was measured with a full-wavelength microplate reader (BioTek Instruments, Inc.).

The ability of HP-OVA to compete with SARS-CoV-2 S (RBD) for ACE2 binding was assessed by a competitive inhibition ELISA as previously described (Li et al., 2010b). Briefly, 1 μg/ml ACE2 protein (Invitrogen, Carlsbad, CA, USA) in 0.01 M Tris buffer (pH 8.8) was coated onto the wells of a polystyrene microplate at 4°C overnight, followed by washing with PBS-T buffer. Then the wells were blocked for 2 h at 37°C with 5% BSA and a mixture of S (RBD) (1 μg/ml) pre-incubated HP-OVA or unmodified OVA at the indicated concentrations was added and incubated. Subsequently, the samples were incubated with an anti-ACE2 antibody (40150-R007, Sino Biological, China) and then detected with an HRP-labeled goat anti-rabbit antibody for 1 h at 37°C. 3,3’,5,5’-Tetramethylbenzidine (TMB), and 1N H_2_SO_4_ were added sequentially. The absorbance at 450 nm was measured by a full-wavelength microplate reader (BioTek Instruments, Inc).

### Cytotoxicity Assay

The cytotoxicity of HP-OVA on different target cells, including Vero E6, Huh 7 and HEK-293T/ACE2 cells, were analyzed by MTT assays (Topscience, Shanghai, China). Briefly, each tested cell lines were seeded into the wells of a 96-well microtiter plate (1 × 10^4^ per well) and incubated at 37°C overnight. Then, HP-OVA or OVA at graded concentrations were added into those cells and incubated at 37°C for 48 h. On the third day post-incubation, 100 μl of DMEM containing MTT [3-(4,5-dimethyl-2-thiazolyl) -2,5-diphenyl-2H-tetrazolium bromide, Sigma Aldrich, St Quentin Fallavier, France] (0.5 mg/ml) was added to equal volumes of cells in wells of 96-well plates and incubated at 37°C for another 4 h. Then, the OD was measured at 570 nm by a full-wavelength microplate reader. Unmodified OVA was used as a negative control. The 50% cytotoxicity concentrations (CC_50_) were calculated using CalcuSyn software.

### Statistical Analysis

Statistical analysis of the experimental data was performed using a one-way ANOVA test in GraphPad Prism 5.0 (San Diego, CA) and represented as means ± SD of at least three measurements. A *p* value of < 0.05 was regarded as statistically significant; the probability level is indicated by single or multiple asterisks (*) (*p < 0.05, **p < 0.01, ***p < 0.001). The percent inhibition and IC_50_ values were calculated using CalcuSyn software.

## Results

### Antiviral Activity of HP-OVA Against SARS-CoV-2 *In Vitro*

**FIGURE 1:**
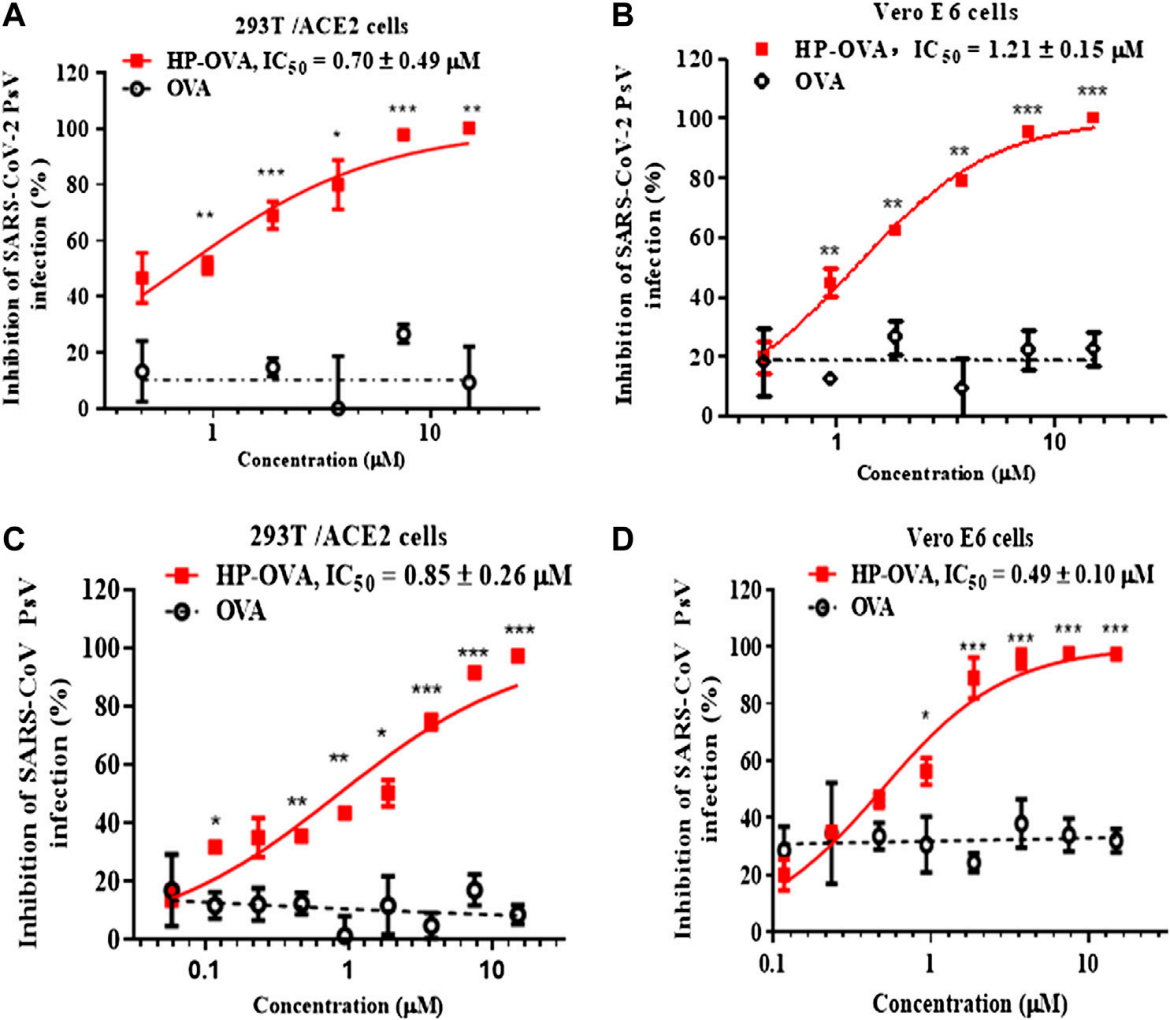
Inhibition of HP-OVA on the infection with SARS-CoV-2 PsV and SARS-CoV PsV. Antiviral activity of HP-OVA against SARS-CoV-2 S PsV infection in 293T/ACE2 **(A)** or Vero E6 **(B)** target cells. Inhibition of single-round infection of SARS-CoV S PsV in 293T/ACE2 **(C)** and Vero E6 **(D)** cells. Data are presented as the mean ± SD of triplicate samples from a representative experiment (*p < 0.05, **p < 0.01, ***p < 0.001).

Our previous studies have shown that OVA can be converted into potent inhibitors through chemical modification with anhydrides to prevent the infection of HIV, HSV-2 and so on (Li et al., 2010a; He et al., 2011; Li et al., 2011). Based on those researches, we try to investigate the antiviral effect of HP-OVA against infection by SARS-CoV-2. At present, pseudovirus (PsV) has become an ideal tool to analyze cell entry of SARS-CoV-2 without safety concerns and possess the morphological characteristics of replication-competent SARS-CoV-2, with the S protein on the envelope membrane. As demonstrated in previous studies (Yin et al., 2018), the pseudotyped system of the SARS-CoV-2 S protein is a classic model that mimics the process of viral entry and studies the interaction of SARS-CoV-2 and host cells. Here, we first utilized SARS-CoV-2 PsV to perform a series of transduction assays. Results showed that HP-OVA exhibited potent inhibitory activity against the entry of SARS-CoV-2 S PsV to the 293T/ACE2 cells (293T cells stably expressing hACE2) in a dose-dependent manner, with an IC_50_ of 0.70 ± 0.49 μM **(** Figure 1A **)**. Notably, the inhibitory activities on Vero E6 cells were consistent with those on ACE2/293T cells, with an IC_50_ of 1.21 ± 0.15 μM, while unmodified OVA had no antiviral activity (Figure 1B). To investigate whether HP-OVA has the same effect on SARS-CoV, which is closely related to SARS-CoV-2 and also employs ACE2 for cell entry, we conducted a pilot experimental test *in vitro* on the anti-SARS-CoV PsV activity using both 293T/ACE2 cells and Vero E6 cells. We found that HP-OVA potently inhibited SARS-CoV infection, with an IC_50_ of approximately 0.85 ± 0.26 μM and 0.49 ± 0.10 μM, respectively (Figures 1C,D).

We next investigated the antiviral activity of HP-OVA against live SARS-CoV-2 *in vitro*. Here, Vero E6 cells were infected with 100 TCID_50_ of live virus and incubated with HP-OVA at different dilution concentrations for 72 h. As shown in Table 1, HP-OVA inhibited the replication of SARS-CoV-2 virus, with an IC_50_ value of 4.78 μM by CPE assay. Additionally, treatment with unmodified OVA showed no inhibitory activity against live SARS-CoV-2. The positive control, remdesivir, potently inhibited virus-induced CPE, with an IC_50_ of 0.65 μM. These antiviral activities indicated that HP-OVA has potent anti-SARS-CoV-2 activity, but the mechanism remains to be explored.

**TABLE 1:** Antiviral activity of HP-OVA against live SARS-CoV-2 in Vero E6 cells^a^.

Compounds	IC_50_ (μM)	IC_90_ (μM)
HP-OVA	4.78 ± 1.03	12.23 ± 2.01
OVA	>50.00	>50.00
Remdesivir	0.65 ± 1.23	2.51 ± 1.41

^a^ The data are presented as mean ± SD of three independent experiments.

### HP-OVA Displays Low Cytotoxicity on the Tested Cell Lines

To evaluate the safety of HP-OVA, target cells including 293T/ACE2, Vero E6 and Huh 7 cells were treated with different concentrations of HP-OVA and assayed by MTT. As shown in Table 2, HP-OVA displayed low cytotoxicity on all tested cell lines, with CC_50_ values ranging from 113.50 to 182.50 μM. The CC_50_ values of HP-OVA were more than 100 times higher than its IC_50_ for inhibiting authentic SARS-CoV-2 and SARS-CoV PsV infection and its selectivity index (SI = CC_50_/IC_50_) ranged from 150.83 to 371.84. Those results indicated that HP-OVA might be safe as an anti-SARS-CoV-2 candidate for use in patients.

**TABLE 2:** Cytotoxicity of HP-OVA *in vitro* ^a^.

Cell lines	HP-OVA	OVA	SI^b^ value of HP-OVA	
	CC_50_ (μM)	CC_50_ (μM)	SARS-CoV-2	SARS-CoV
Vero E6 cells	182.50 ± 29.00	>200.00	150.83	214.71
ACE2/293T cells	182.20 ± 59.75	>200.00	260.29	371.84
Huh 7 cells	113.50 ± 23.36	>200.00	Not done	Not done

^a^ The data are presented as mean ± SD of three independent experiments.

^b^ SI, selectivity index = CC_50_/IC_50_.

### HP-OVA Inhibited SARS-CoV-2 Through Inhibiting S Protein-Mediated Cell-Cell Fusion

**FIGURE 2:**
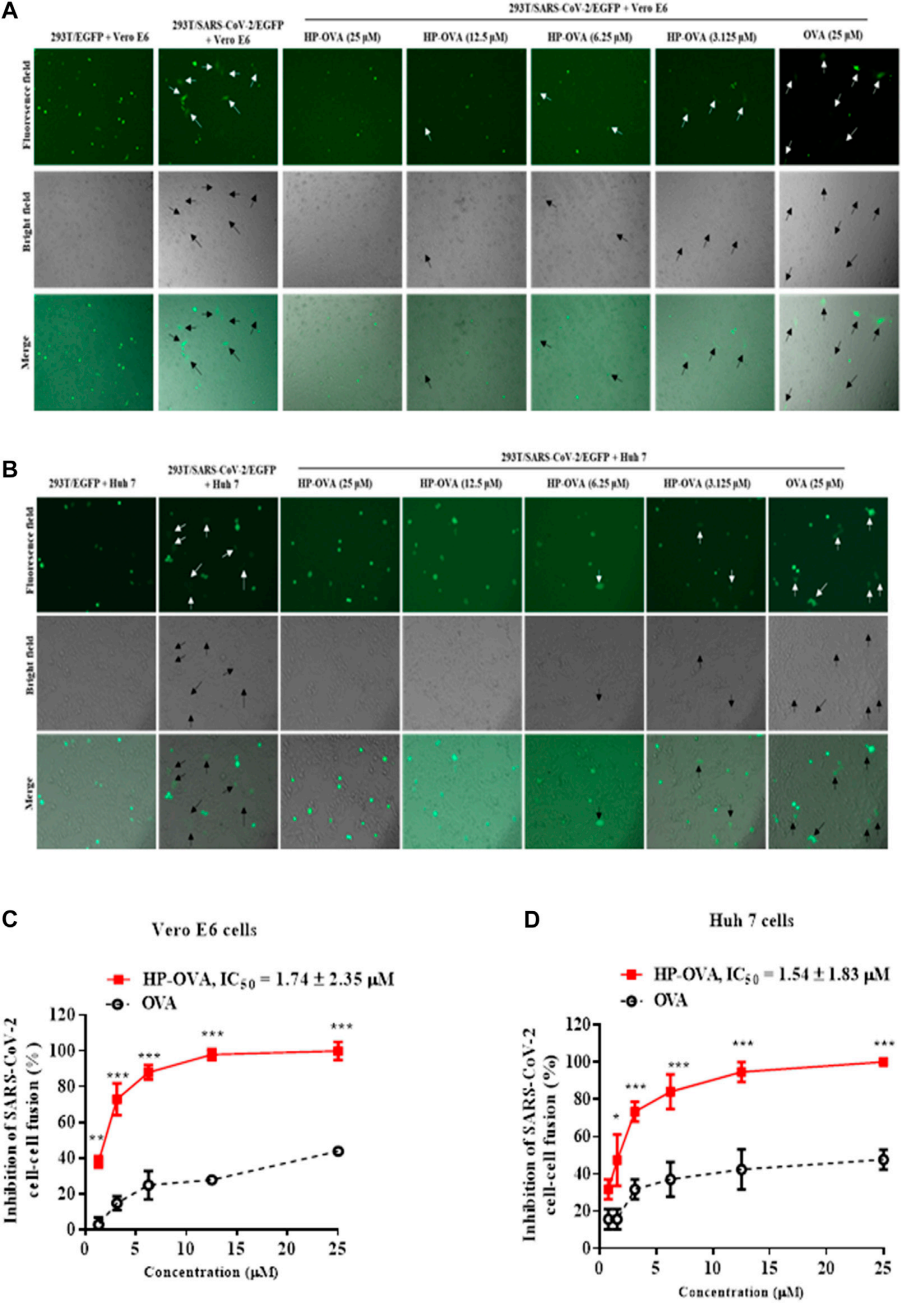
Inhibitory activity of HP-OVA against SARS-CoV-2 S-mediated cell-cell fusion. Images were captured at 12 h after treatment with HP-OVA or OVA on SARS-CoV-2 S protein-mediated cell-cell fusion. The syncytia of Vero E6 cells **(A)** or Huh 7 cells **(B)** and HEK293T cells with SARS-CoV-2 overexpression are marked in the pictures. Representative results from three fields were selected randomly from each sample with scale bars of 50 μm **(C, D)** The number of syncytia was counted under an inverted fluorescence microscope, and the percentage of inhibition was calculated as described in the Methods. Data are presented as the mean ± SD of triplicate samples from a representative experiment (*p < 0.05, **p < 0.01, ***p < 0.001).

The spike (S) glycoprotein of SARS-CoV-2 binds ACE2 cellular receptors to facilitate fusion and ultimately entry into cells. Therefore, we herein analyzed the potential role of HP-OVA on SARS-CoV-2 S-mediated cell-cell fusion. In this widely adopted cell-cell fusion system, SARS-CoV-2 S and green fluorescent protein genes were transfected into HEK-293T cells. In a syncytium-formation assay, the size of a syncytium is usually ≥ 2-fold larger than that of a normal cell, and the numbers of syncytia and fluorescence-labeled fused cells were counted under an inverted microscope. Here, we chose two kinds of cells expressing hACE2 receptor as the target cells including Vero E6 (Figure 2A) and Huh 7 cells (Figure 2B). As shown in Figure 2A, HP-OVA significantly inhibited S-mediated 293T/SARS-CoV-2/EGFP and Vero E6 cell-cell fusion, resulting in the reduction in syncytium formation in a dose-dependent manner, with an IC_50_ of 1.74 μM (Figure 2C). Correspondingly, HP-OVA showed potent fusion inhibitory activity on SARS-CoV-2 S-mediated 293T/SARS-CoV-2/EGFP and Huh 7 cell-cell fusion, with an IC_50_ of 1.54 μM (Figures 2B,D). It is worth noting that unmodified OVA showed no inhibitory activity at concentrations up to 25 μM in cell-cell fusion assays. These results suggest that HP-OVA exhibits inhibitory activity against SARS-CoV-2 by blocking S-mediated cell-cell fusion.

### The Antiviral Activity of HP-OVA Was Attributed to the Disruption of the S Protein-ACE2 Interaction

**FIGURE 3:**
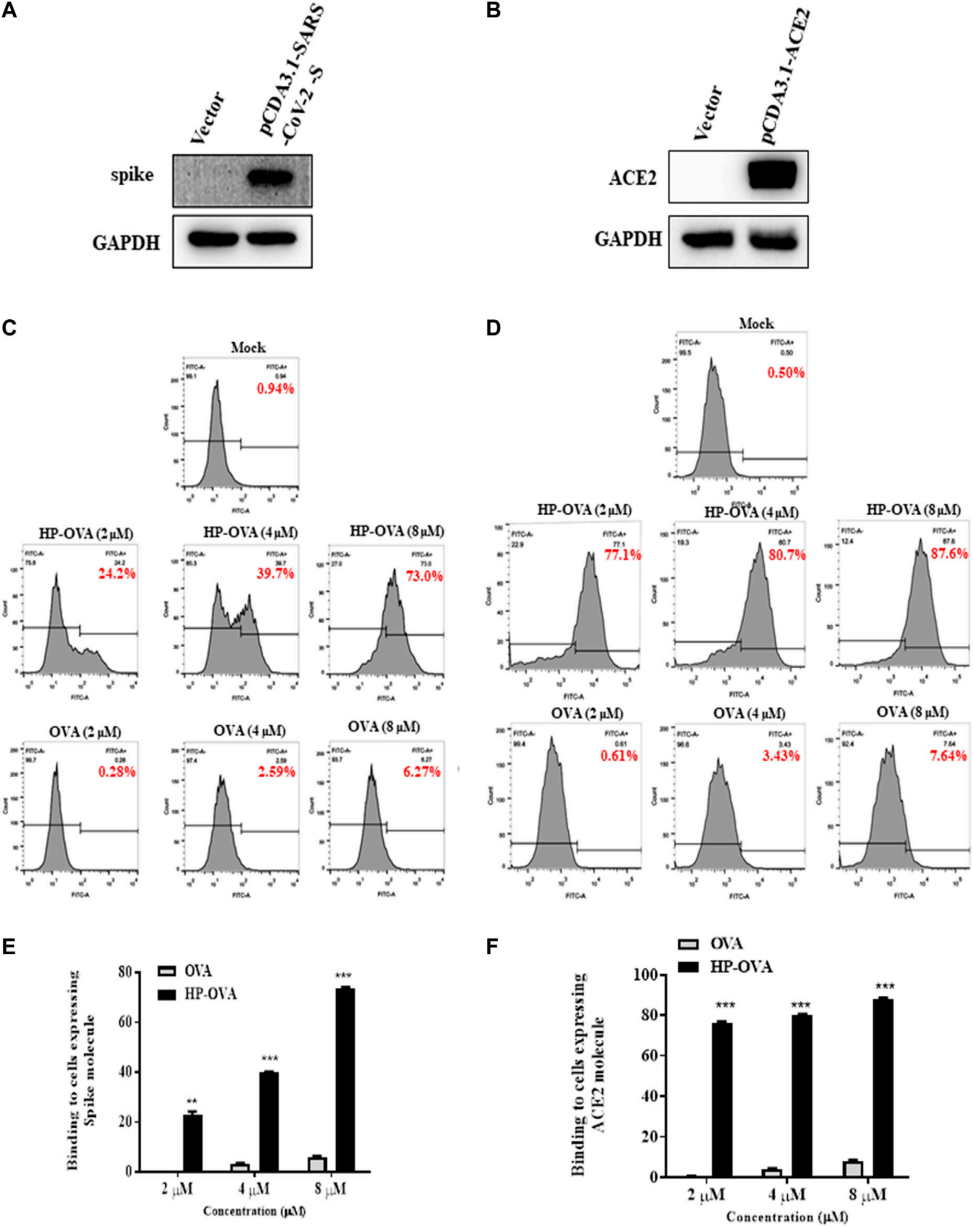
HP-OVA binding to both SARS-CoV-2 S and ACE2 protein. Analysis of the expression of SARS-CoV-2 S **(A)** and ACE2 **(B)** in HEK-293T cells by western blot. The binding of HP-OVA to cells expressing SARS-CoV-2 S **(C)** or ACE2 **(D)** was assessed by flow cytometry. A representative flow histogram and quantification of the binding of HP-OVA to cells expressing SARS-CoV-2 S **(E)** or ACE2 **(F)** were shown. Data are presented as the mean ± SD (*p < 0.05, **p < 0.01, ***p < 0.001).

**FIGURE 4:**
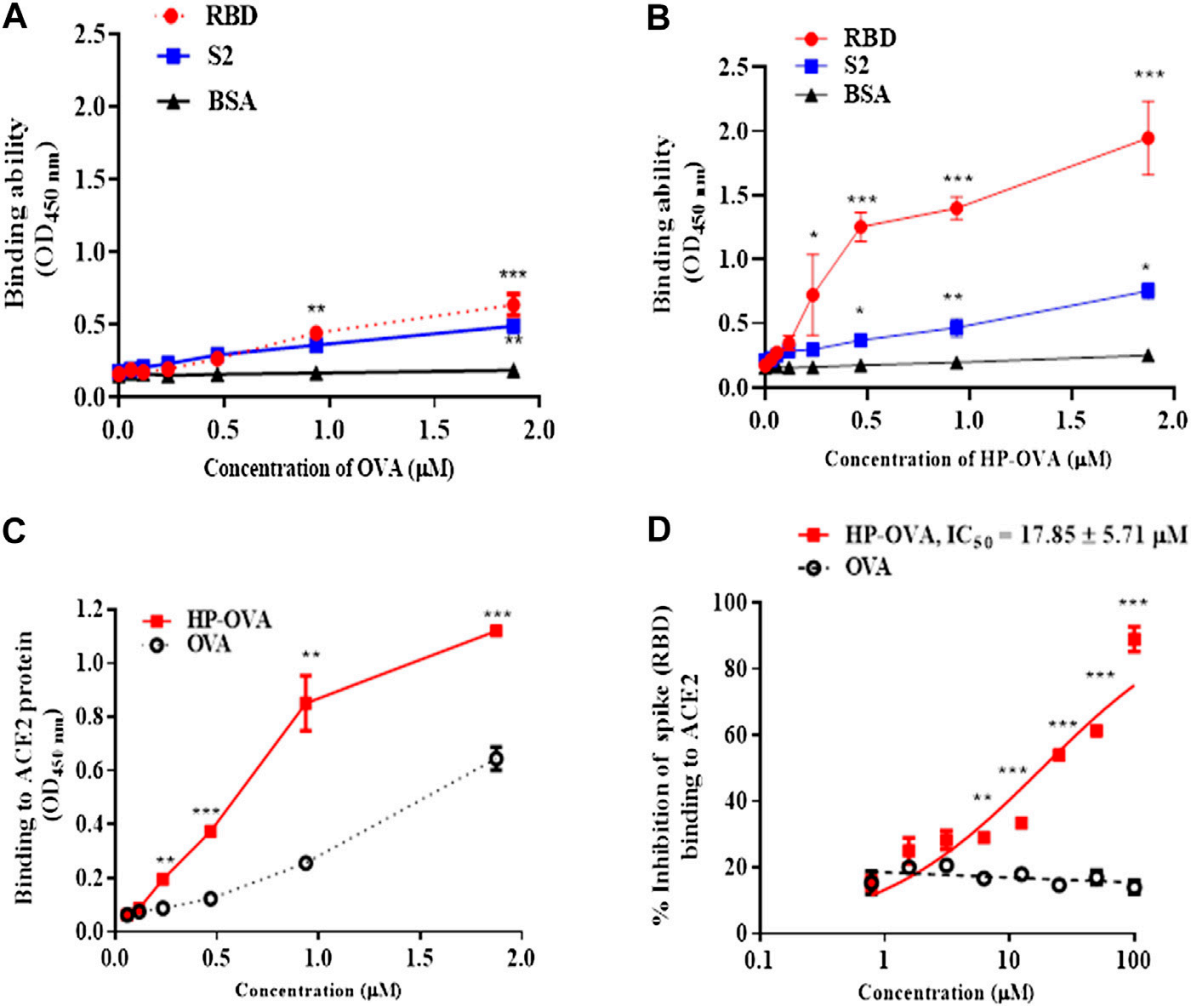
The interaction of HP-OVA with SARS-CoV-2 S and ACE2. The binding of OVA to SARS-CoV-2 spike (RBD), S2 and BSA protein was assessed by ELISA **(A)**. The binding of HP-OVA to SARS-CoV-2 spike (RBD), S2 and a negative control BSA protein was assessed by ELISA **(B)**. The binding ability of HP-OVA to ACE2 protein was assessed by ELISA **(C)**. Inhibition of the interaction between spike (RBD) and ACE2 proteins by HP-OVA, as determined by a competitive inhibition ELISA **(D)**. Data are presented as the mean ± SD of triplicate samples from a representative experiment (*p < 0.05, **p < 0.01, ***p < 0.001).

The SARS-CoV-2-S/ACE2 interface was found to be a key determinant of SARS-CoV-2 transmissibility. Our preliminary work have revealed that HP-OVA is highly effective against SARS-CoV-2 S-mediated cell-cell fusion and SARS-CoV-2 S PsV infection, suggesting that HP-OVA might be a viral entry inhibitor by interacting with either the S protein of coronaviruses or ACE2 receptor on the target cellular surface. To investigate this hypothesis, SARS-CoV-2 S (Figure 3A) or ACE2 (Figure 3B) was transiently overexpressed in HEK-293T cells, and flow cytometry was used to analyze the binding activity. As shown in Figures 3C–F, HP-OVA notably bound to HEK-293T cells overexpressing both S and ACE2 proteins in a dose-dependent manner, while unmodified OVA showed no corresponding effect. To further confirm the specific targets, the binding of HP-OVA to S or ACE2 molecules was subsequently determined by ELISA. The results also showed that HP-OVA could bind to both the S (RBD) protein (Figure 4B) and ACE2 protein (Figure 4C) in a dose-dependent manner. We further determined the binding of HP-OVA to the spike S2 protein of SARS-CoV-2 by ELISA. As shown in Figure 4B, HP-OVA could also bind to S2 protein, while the binding ability to S2 proteins is weaker than RBD protein. In addition, we found that HP-OVA could not bind to an irrelevant coating protein antigen control BSA, indicating to the specific binding to both S and ACE2 proteins (Figure 4B). These results indicated that HP-OVA inhibits SARS-CoV-2-mediated viral entry at the cell surface attachment step by directly interacting with S protein and ACE2 receptor.

To determine whether the potential effect of HP-OVA on the interaction between S protein and ACE2 receptor, a competitive inhibition assay was conducted by ELISA. As shown in Figure 4D, HP-OVA significantly inhibited the binding of S and ACE2 in a dose-dependent manner, with an IC_50_ of 17.85 μM. These results indicated that HP-OVA may bind to both S protein and ACE2 receptor and then interfere with their interaction, resulting in the inhibition of viral entry.

## Discussion

Currently, the rapid spread of COVID-19 has resulted in an urgent requirement for effective therapeutic strategies against SARS-CoV-2. Initially, without licensed vaccines or approved antiviral drugs, COVID-19 treatment was mainly based on the experience of clinicians. Nonspecific antiviral drugs, including IFN-α (recombinant human IFN-α1b, IFN-α2a), lopinavir/ritonavir (Aluvia, HIV protease inhibitors), chloroquine phosphate, favipiravir and ribavirin, have been clinically used as antiviral therapies according to the National Health Commission (NHC) of the People's Republic of China (Huang et al., 2020; Lu, 2020). To date, many potential drugs have been expected to have therapeutic potential, including inhibition of TMPRSS2 (Hoffmann et al., 2020) (i.e., camostat mesylate, nafamostat, loprazolam, and rubitecan) and antiviral drugs inhibiting viral RdRp (i.e., remdesivir, and favipiravir) (Elfiky, 2020; Lung et al., 2020) and 3CLpro (i.e., poziotinib, fostamatinib, ziprasidone, and telcagepant) (Jo et al., 2020; Ul Qamar et al., 2020) as well as virus/host cell membrane fusion (i.e., EK1C4, nelfinavir mesylate, and IBP02) (Hoffmann et al., 2020; Xia et al., 2020; Zhu et al., 2020). However, the efficacies *in vivo* still require further confirmation, and their potential use for the treatment of infection by other coronaviruses and emerging coronaviruses in the future is unclear. Therefore, drug development for treating COVID-19 is timely and important due to its rapid expansion.

Viral entry inhibitors have proven effectiveness and safety for the treatment of viral infections, and targeting viral entry may have a greater potential in the development of pan-CoV inhibitors for future coronavirus outbreaks (Chu et al., 2008; Sun et al., 2013; Xia et al., 2014; Li et al., 2017; Pu et al., 2019). Combined with our previous studies, we focused on HP-OVA because chicken OVA is the main protein in egg white, making up 60–65% of the total protein. Second, HP-OVA is convenient to synthesize by anhydride modification with OVA, which is isolated from chicken eggs and less expensive than RSA, which is purified from animal sera. Third, HP-OVA exerts a broad-spectrum effect on a series of HIV strains by blocking HIV entry. Our research demonstrated that HP-OVA could inhibit Vero E6 cell infection with live SARS-CoV-2, with an IC_50_ value of 4.78 μM and an IC_90_ value of 12.23 μM. Furthermore, HP-OVA obviously inhibited pseudotyped SARS-CoV-2 entry into two different target cells, with an IC_50_ value of 0.70 and 1.21 μM, respectively. Notably, HP-OVA also showed inhibitory activity against SARS-CoV infection of 293T/ACE2 and Vero E6 cells, with an IC_50_ value of 0.85 and 0.49 μM, respectively. Furthermore, our results showed that HP-OVA displayed low cytotoxicity on all tested cell lines, with CC_50_ values ranging from 113.50 to 182.50 μM. The CC_50_ values of HP-OVA were more than 100 times higher than its IC_50_ for inhibiting authentic SARS-CoV-2 PsV infection and its SI values ranged from 150.83 to 260.29, indicating that HP-OVA might be safe as an anti-SARS-CoV-2 candidate for use in patients. Therefore, this study suggests that HP-OVA has broad-spectrum antiviral activity by inhibiting viral entry, and it can be used for the treatment and prevention of infection by not only SARS-CoV-2 but also other human coronaviruses (HCoVs).

It is worth mentioning that OVA is a commonly used as an antigen for vaccination experiments and immunization researches. One may raise a concern about the potential of HP-OVA to induce immune responses when it is used as a nasal spray. However, several studies have reported that mucosal immunization by topical administration with soluble proteins, including OVA, without any adjuvants, are usually unable to induce strong local immune responses (Staats et al., 1994; Walker, 1994; Di Tommaso et al., 1996). Our previous studies have also certified that HP-OVA has no harmful or deleterious impact on the function of immune cells (Li et al., 2010a). Actually, anhydride-modiβed proteins, such as anhydride-modiβed bovine β-lactoglobulin, have been studied and utilized as microbicides against HIV and HPV in clinics for years, and their effectiveness and safety as drugs have been veriβed (Neurath et al., 1996; Guo et al., 2016; Hua et al., 2019).

Another important problem for development of HP-OVA as an antiviral agent is to confirm its *in vivo* therapeutic efficacy of HP-OVA against authentic SARS-CoV-2 infection in animal models. To date, various species have been used as animal models of SARS-CoV-2 infection, including hACE2 transgenic mice, African green monkey, Baboon, Cynomolgus macaque, and Ferret and Syrian hamster. However, there is currently no single, simple and optimal animal models for SARS-CoV-2 infection (Khoury et al., 2020; Muñoz-Fontela et al., 2020). In additional, there are several significant differences between the pathogenesis and kinetic of human infection and animal models. Furthermore, it is also not clear which is the best outcome metric to study-for example, should an intervention aim to reduce the viral titer, pathology or lethality? The most suitable animal model and outcome measure for a particular application depends on the therapeutic intention, as well as the cost, timing and availability. Taken all consideration, we have not verified the antiviral effectiveness of HP-OVA against SARS-CoV-2 infection on animal models. The next stage of assessing HP-OVA’s efficacy will be typically involved animal testing, which is extremely important and will strengthen our findings.

The S protein interaction with ACE2 on the host cell cytoplasmic membrane initiates viral infection. Strategies capable of disrupting the S protein interaction with ACE2 could be of significant therapeutic value and could contribute to/favor the resolution of the pandemic that is developing worldwide because the binding affinity of the SARS-CoV-2 S protein to ACE2 is 10 to20-fold higher than that of the S protein of SARS-CoV, which may contribute to the higher contagiousness of SARS-CoV-2 than SARS-CoV (Shang et al., 2020; Walls et al., 2020). Our preliminary results indicated that HP-OVA could bind to both ACE2 and the S protein (RBD domain) directly. In addition, HP-OVA interferes to the interaction between SARS-CoV-2 S protein and ACE2 receptor on the cell surface, leading to the inhibition of SARS-CoV-2 infection and S protein-mediated cell-cell fusion. The unmodified OVA protein can not interfere to the binding of S protein and ACE2 receptor. Our previously study reported that the binding ability of HP-OVA is closely correlated with the number of the positively charged side chains of lysine and arginine residues were converted to negatively charged side chains after modiβcation by HP (Li et al., 2010a). Thus, the positively charged side chains of HP-OVA might account for the antiviral activity of HP-OVA since unmodiβed OVA did not showed either an afβnity of binding to ACE2 or S protein (RBD domain), as well as the inhibitory activity against SARS-CoV-2 infection.

Our results showed HP-OVA could also bind to S2 protein, while the binding ability to S2 protein is weaker than RBD protein. Indeed, there is less enthusiasm for developing HP-OVA as a specific antiviral entry inhibitor because it can bind to a variety of viral membrane proteins. Our previous studies have certified that anhydride-modified proteins could inhibit several kinds of viruses, including HIV, HPV, RSV and MERS-CoV. It is worth mentioning that the specific antiviral inhibitors are only effective against SARS-CoV-2, whereas the non-specific antiviral agents may also be effective against other pathogens, such as SRAS or other coronavirus. The preliminary results indicated that HP-OVA was effective against SARS-CoV infection, suggesting that it has good potential to be developed as a promising active component for prevention of multiple coronavirus diseases.

Since HP-OVA can bind to ACE2 receptor and ACE2 helps modulate the many activities of angiotensin Ⅱ (ANG Ⅱ) that increases blood pressure and inflammation, increasing damage to blood vessel linings and various types of tissue injury. Therefore, the potential effect of HP-OVA on ACE2 is warranted. Another problem for development of chemically modified OVA as pan-CoV inhibitor-based therapeutic and prophylactic for the treatment and prevention of the current COVID-19 pandemic is the potential risk of causing side effects in people who are allergy to egg protein (Honma et al., 1996). Fortunately, egg allergy occurs seldom in adults, but mostly in young children (less than five years old) (Mine and Yang, 2008). Therefore, we expect that there will be only very few adults with egg allergy, and those people should be excluded from the clinical trials of HP-OVA-based microbicide.

**FIGURE 5:**
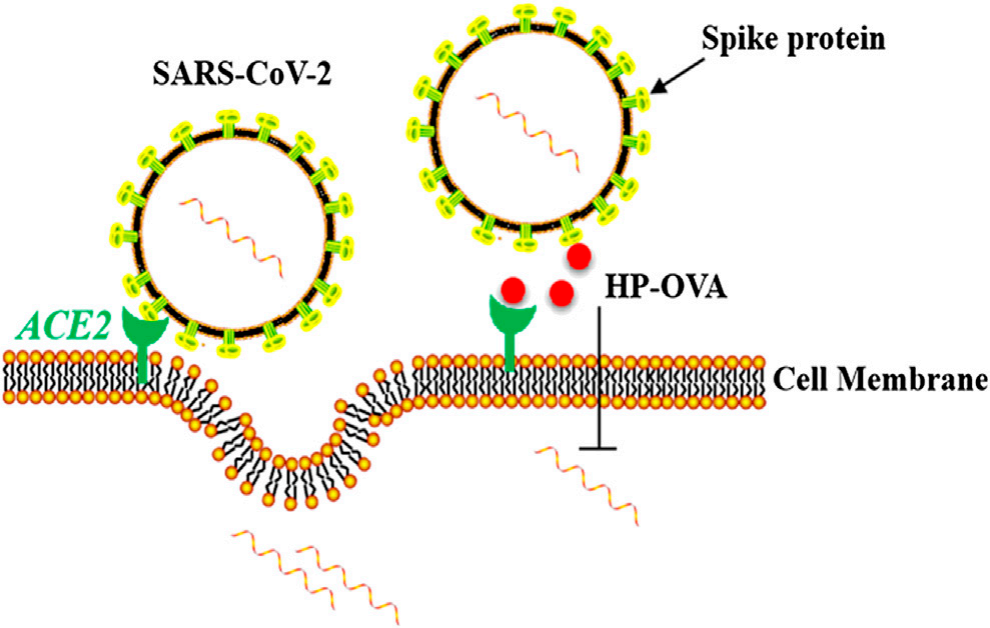
Schematic representation of the molecular mechanisms of HP-OVA against SARS-CoV-2 infection. HP-OVA binds to both the S protein of SARS-CoV-2 and host angiotensin-converting enzyme 2 (ACE2), the functional receptor of SARS-CoV-2, and disrupts the S protein-ACE2 interaction, thereby exhibiting inhibitory activity against SARS-CoV-2 infection.

Taken all consideration, HP-OVA can be more easily produced on a large scale and are more cost-effective than neutralizing antibodies and other large protein-based inhibitors, thus we believe HP-OVA is a promising candidate for optimization and development as a pan-CoV inhibitor-based therapeutic and prophylactic for the treatment and prevention of the current COVID-19 pandemic and may help in the future to prevent new viruses that have an affinity between the S protein and ACE2 receptor. The mechanism of action of HP-OVA against SARS-CoV-2 infection was shown in Figure 5.

## Data Availability Statement

The original contributions presented in the study are included in the article, further inquiries can be directed to the Corresponding authors.

## Author Contributions

TL contributed to perform all the experiments, analyze the data and draft the manuscript. JQ, XN, and QM helped with experimental design and manuscript writing. CZ, and PC performed some of the experiments. QZ and MC contributed to data analysis. ZY, SL, and LL supervised the study, edited and reviewed the manuscript. All authors read and approved the final manuscript for publication.

## Funding

This study was supported by the Natural Science Foundation of China (82073896 and 81673481 to LL), the Zhejiang University special scientific research fund for COVID-19 prevention and control to LL, the Opening Project of Zhejiang Provincial Preponderant and Characteristic Subject of Key University (Traditional Chinese Pharmacology) Zhejiang Chinese Medical University (ZYAOXZD2019001 to LL), Guangdong Basic and Applied Basic Research Foundation (2019A1515010061 to LL), the Major Scientific and Technological Project of Guangdong Province (2019B020202002 to SL), China Evergrande Group, Jack Ma Foundation (2020-CMKYGG-02 to ZY) and Postdoctoral Science Foundation of Zhejiang Province to JQ.

## Conflict of Interest

The authors declare that the research was conducted in the absence of any commercial or financial relationships that could be construed as a potential conflict of interest.
